# Auditory Verbal Hallucinations in Schizophrenia and Bipolar Disorders

**DOI:** 10.1192/j.eurpsy.2025.2060

**Published:** 2025-08-26

**Authors:** J.-E. Yttri, A. Urfer-Parnas, S. M. Arnfred, J. Parnas

**Affiliations:** 1 Psychiatry Vest, Mental Health Service Region Zealand, Slagelse; 2 Faculty of health and medical sciences, University of Copenhagen; 3 Psychiatry in the Capital Region of Denmark, Mental Health Centre Amager; 4 Psychiatry in the Capital Region of Denmark, Mental Health Center Glostrup; 5 University of Copenhagen, Center for Subjectivity Research, Copenhagen, Denmark

## Abstract

**Introduction:**

Auditory verbal hallucinations (AVH) are prevalent in schizophrenia but also occur in bipolar disorder, yet differences in their phenomenology remain poorly understood. This ongoing study aims to fill this gap by comparing the phenomenological characteristics of AVH in schizophrenia and bipolar disorder.

**Objectives:**

The research questions we are interested in are:

- The nature of hallucinations. We hypothesize that hallucinatory experiences in bipolar disorders is connected to the mood, for example mania.
- We hypothesize that auditive verbal hallucinations in bipolar disorders will not have the immanent character as in schizophrenia and won’t be experienced in a pathologically altered private space.
- We hypothesize that bipolar patients after a remission from the episode will consider their voices as a sign of disease whereas the patients with schizophrenia will continue to ascribe extraordinary meanings to their hallucinations.

**Methods:**

Building on our prior research in schizophrenia, we are conducting semi-structured, phenomenologically oriented qualitative interviews with patients diagnosed with bipolar disorder who experience AVH. Participants are recruited from outpatient clinics and hospital wards in Denmark. Data will be analyzed using thematic analysis with a bottom-up approach.

**Results:**

Preliminary findings from our ongoing study will be presented.

**Conclusions:**

A deeper understanding of AVH phenomenology in schizophrenia and bipolar disorder can lead to more accurate differential diagnoses, reducing the risk of misdiagnosis and ensuring that patients receive appropriate and timely treatment.

**Disclosure of Interest:**

None Declared

